# SARS-CoV-2 RBD protein enhances the oncolytic activity of the vesicular stomatitis virus

**DOI:** 10.3389/fimmu.2023.1082191

**Published:** 2023-01-31

**Authors:** Almohanad A. Alkayyal, Reham Ajina, Marco Cacciabue, Aaesha A. Alkayyal, Nizar H. Saeedi, Taofik Hussain Alshehry, Feras Kaboha, Mohammed A. Alotaibi, Nada Zaidan, Khalid Shah, Fayhan Alroqi, Ahmad Bakur Mahmoud

**Affiliations:** ^1^ Department of Medical Laboratory Technology, Faculty of Applied Medical Sciences, University of Tabuk, Tabuk, Saudi Arabia; ^2^ Immunology Research Program, King Abdullah International Medical Research Center, Riyadh, Saudi Arabia; ^3^ Department of Clinical Laboratory Sciences, College of Applied Medical Sciences, King Saud bin Abdulaziz University for Health Sciences, Riyadh, Saudi Arabia; ^4^ Instituto de Agrobiotecnología y Biología Molecular (IABIMO), Instituto Nacional de Tecnología Agropecuaria (INTA), Consejo Nacional de Investigaciones Científicas y Técnicas (CONICET), De los Reseros y N. Repetto s/n, Hurlingham, Buenos Aires, Argentina; ^5^ Departamento de Ciencias Básicas, Universidad Nacional de Luján, Luján, Buenos Aires, Argentina; ^6^ College of Medicine, Taibah University, Almadinah Almunwarah, Saudi Arabia; ^7^ King Abdullah International Medical Research Centre, King Saud University for Health Sciences, Ministry of National Guard Health Affairs, Jeddah, Saudi Arabia; ^8^ King Abdulaziz City for Science and Technology-Brigham and Women's Hospital (KACST-BWH) Centre of Excellence for Biomedicine, Joint Centers of Excellence Program, King Abdulaziz City for Science and Technology (KACST), Riyadh, Saudi Arabia; ^9^ Center for Stem Cell and Translational Immunotherapy (CSTI), Brigham and Women’s Hospital, Harvard Medical School, Boston, MA, United States; ^10^ Department of Neurosurgery, Brigham and Women’s Hospital, Harvard Medical School, Boston, MA, United States; ^11^ Harvard Stem Cell Institute, Harvard University, Cambridge, MA, United States; ^12^ Department of Immunology, Ministry of the National Guard - Health Affairs, Riyadh, Saudi Arabia; ^13^ Faculty of Medicine, King Saud bin Abdulaziz University for Health Sciences, Riyadh, Saudi Arabia; ^14^ College of Applied Medical Sciences, Taibah University, Madinah, Saudi Arabia; ^15^ Strategic Research and Innovation Laboratories, Taibah University, Madinah, Saudi Arabia; ^16^ Immunology Research Program, King Abdullah International Medical Research Center, Jeddah, Saudi Arabia

**Keywords:** SARS-CoV-2 RBD, oncolytic virotherapy, VSV-Δ51 enhancement, VSV-Δ51 production, B16F10 melanoma models

## Abstract

Despite recent advances in the research on oncolytic viruses (OVs), a better understanding of how to enhance their replication is key to improving their therapeutic index. Understanding viral replication is important to improve treatment outcomes based on enhanced viral spreading within the tumor milieu. The VSV-Δ51 oncolytic virus has been widely used as an anticancer agent with a high selectivity profile. In this study, we examined the role of the SARS-CoV-2 spike protein receptor-binding domain (RBD) in enhancing VSV-Δ51 viral production and oncolytic activity. To test this hypothesis, we first generated a novel VSV-Δ51 mutant that encoded the SARS-COV-2 RBD and compared viral spreading and viral yield between VSV-Δ51-RBD and VSV-Δ51 *in vitro*. Using the viral plaque assay, we demonstrated that the presence of the SARS-CoV-2 RBD in the VSV-Δ51 genome is associated with a significantly larger viral plaque surface area and significantly higher virus titers. Subsequently, using an ATP release-based assay, we demonstrated that the SARS-CoV-2 RBD could enhance VSV-Δ51 oncolytic activity *in vitro*. This observation was further supported using the B16F10 tumor model. These findings highlighted a novel use of the SARS-CoV-2 RBD as an anticancer agent.

## Introduction

Oncolytic virus (OV) therapy is a novel cancer treatment strategy. It involves a wide range of weakly pathogenic wild-type and genetically modified viruses that preferentially replicate in and specifically kill malignant cells (1–3). Several OVs have shown promising antitumor responses in preclinical and clinical settings, including rhabdoviruses such as Maraba (4, 5) and vesicular stomatitis virus (VSV) (6, 7). VSV has significant potency compared to other viruses due to its extremely rapid life cycle. Indeed, VSV viral progenies are detectable as early as six hours after infection. Additionally, VSV is relatively safe in humans compared to many other viral species (8). These characteristics make VSV an ideal platform to be utilized as a gene delivery vector.

VSV has been widely used as a recombinant vaccine to treat cancer and to protect against viral infections (9), including Ebola virus (EBOV) (10), respiratory syncytial virus (RSV) (11), human immunodeficiency virus (HIV) (12), and severe acute respiratory syndrome coronavirus 1 (SARS-CoV-1) (13). This utility has been attributed to its ability to generate humoral immunity and cellular immunity to the foreign antigens that are expressed. More recently, VSV vectors have also been utilized as a SARS-CoV-2 vaccine platform in a global effort to combat the coronavirus disease responsible for the 2019 pandemic (COVID-19). Most of these studies evaluated the efficacy of the VSV vaccine by encoding either the full sequence or part of the sequence of the SARS-CoV-2 spike proteins, including S1 and the receptor-binding domain (RBD) (14, 15).

VSV-Δ51 is an engineered mutant of the VSV virus that harbors a deletion of methionine (M) 51. Deletion of M51 renders the VSV-Δ51 virus more sensitive to the interferon (IFN) response in normal cells. Because cancer cells are typically characterized by a diminished response to interferon, the VSV-Δ51 virus can selectively infect tumor cells. Hence, VSV-Δ51 is less toxic than wild-type VSV, and thus, it has a significantly improved therapeutic index (16, 17). The current study explored the novel use of the SARS-CoV-2 RBD to enhance VSV-Δ51 viral production and oncolytic activity.

## Results

### The SARS-CoV-2 RBD induces VSV-Δ51 viral spreading in VERO cells

**Figure 1:**
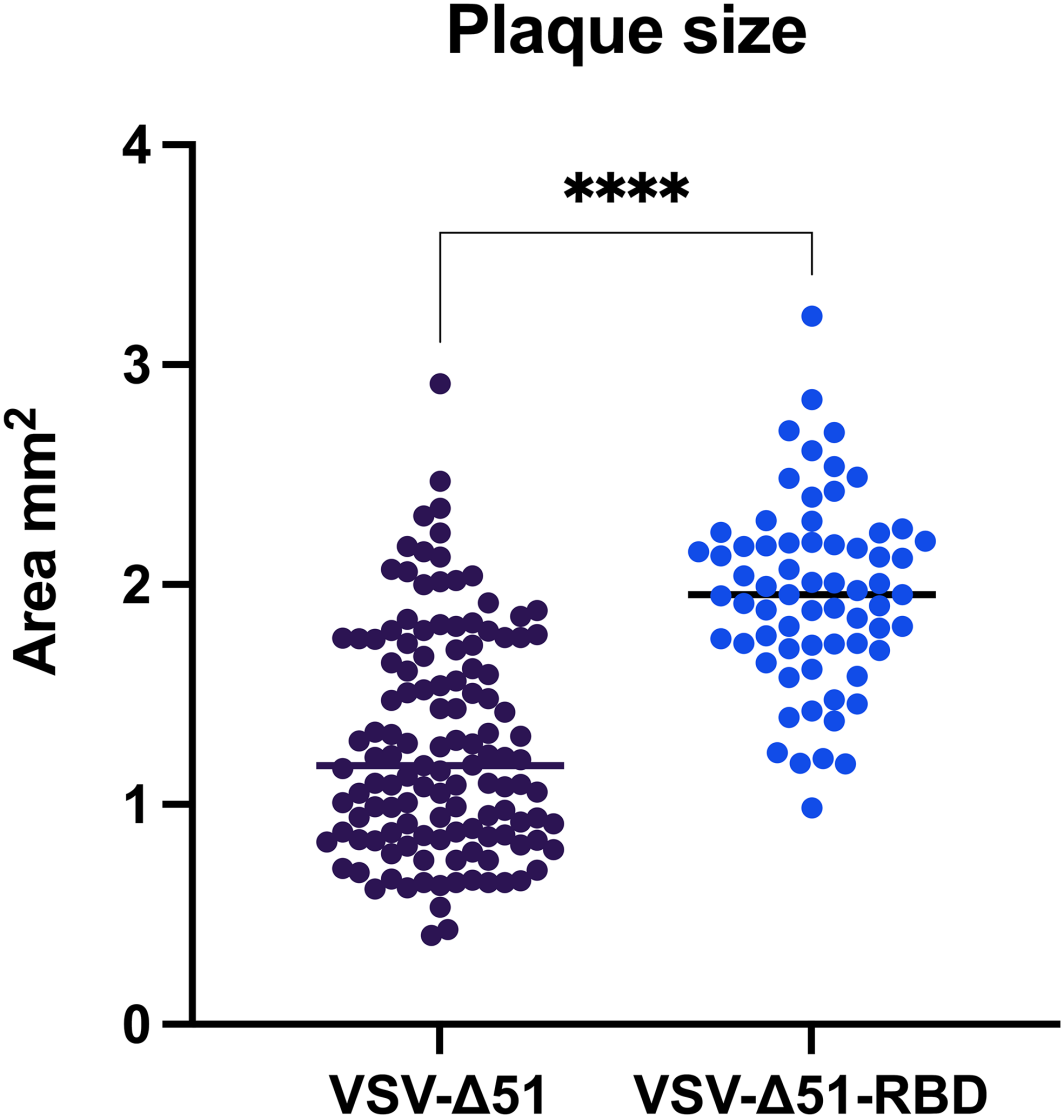
The RBD induces VSV-Δ51 viral spreading in VERO cells: Surface area of virus-formed plaques in VERO cells infected with VSV-Δ51 or VSV-Δ51-RBD viruses. ****P < 0.00005, by t-test.

Because VSV-Δ51 has been documented as a potent oncolytic virus with a short replication lifecycle, we wanted to determine whether the incorporation of the SARS-CoV-2 RBD gene into the VSV-Δ51 viral genome would improve the viral spreading capacity. After we confirmed the expression of the RBD protein by Western blot (our unpublished data), a conventional viral plaque assay of VSV-Δ51 or VSV-Δ51-RBD viruses in VERO cells was carried out, and the size of the viral plaques were measured using ViralPlaque. Surprisingly, the surface area of VSV-Δ51-RBD plaques was significantly larger than that of the plaques formed by the VSV-Δ51 virus, with a mean surface area of 1.962 mm^2^ vs. 1.279 mm^2^, respectively (**Figure 1**). This observation suggests that the SARS-CoV-2 RBD favors viral spreading.

### The SARS-CoV-2 RBD enhances VSV-Δ51 viral yield in VERO cell line

**Figure 2:**
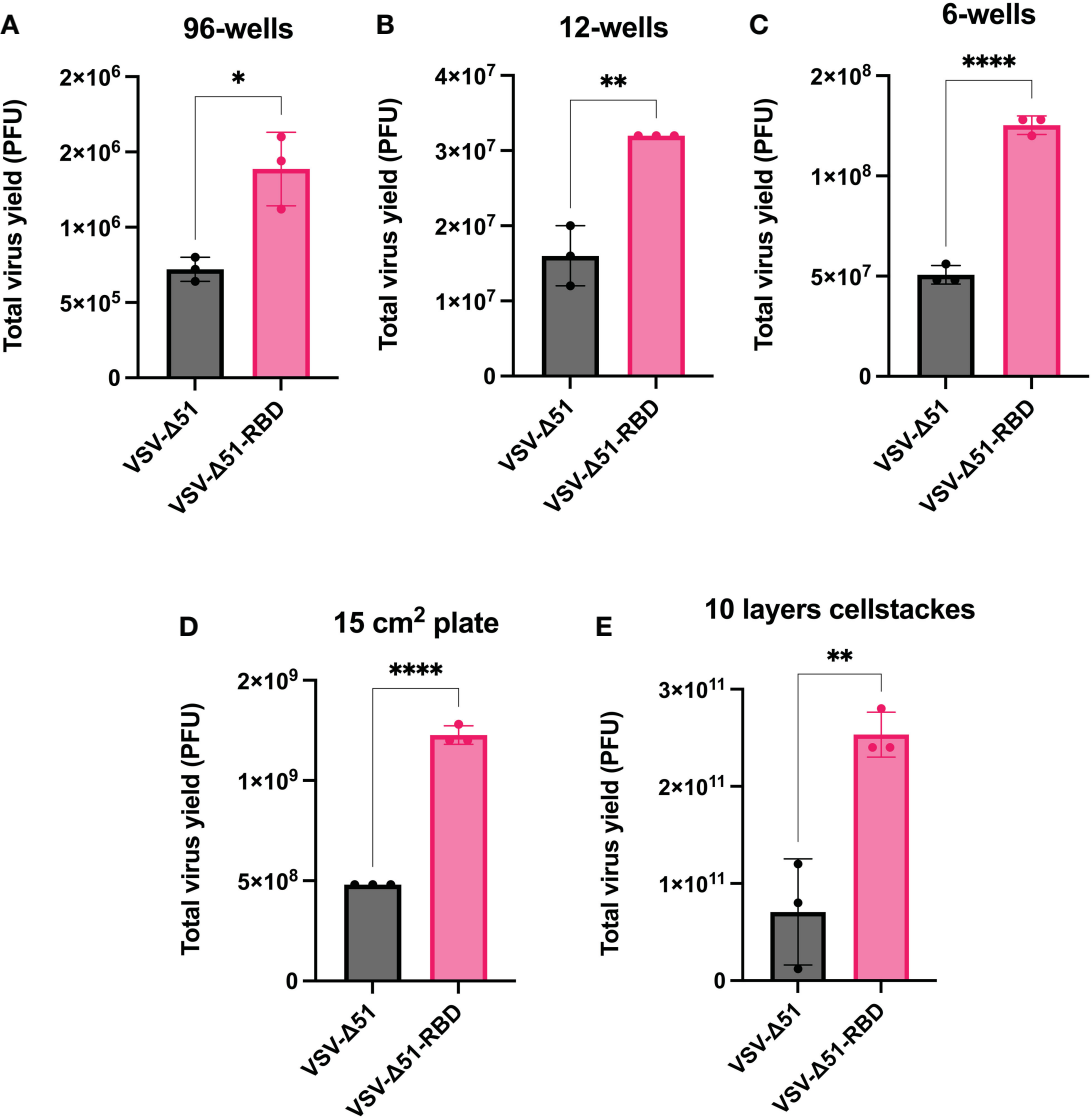
The RBD expression in the VSV-Δ51 virus increases the viral yield: Virus yields from VERO cells infected with either VSV-Δ51 or VSV-Δ51-RBD at an MOI of 0.1 for 24 hours in **(A)** a 96-well format, **(B)** a 12-well format, **(C)** a 6-well format, **(D)** 15 cm plates or **(E)** 10 layers of cellstacks. P < 0.05, *P < 0.05, **P < 0.005, ****P < 0.00005, by t test.

To determine whether the enhanced viral spreading would be translated to an increase in viral yield, we evaluated the total virus yield from VERO cells infected with either VSV-Δ51 or VSV-Δ51-RBD viruses. We measured the virus yield at different tissue culture scales ranging from 96 wells, with a surface area of 0.32 cm^2^, to 10 layers of cellSTACKs, with a surface area of 6,350 cm^2^. As expected, VSV-Δ51-RBD-infected VERO cells had significantly higher virus titers than VSV-Δ51-infected VERO cells across all of the different culture scales as follows: 96-well format, 12-well format, 6-well format, 15-cm plates and 10 layers of cellSTACKs (P < 0.05, *P < 0.05, **P < 0.005, ****P < 0.00005, respectively) (**Figure 2**). This finding suggests that the SARS-CoV-2 RBD could be utilized to improve viral yields for large-scale virus production.

### The SARS-CoV-2 RBD enhances VSV-Δ51 oncolytic activity *in vitro*

**Figure 3:**
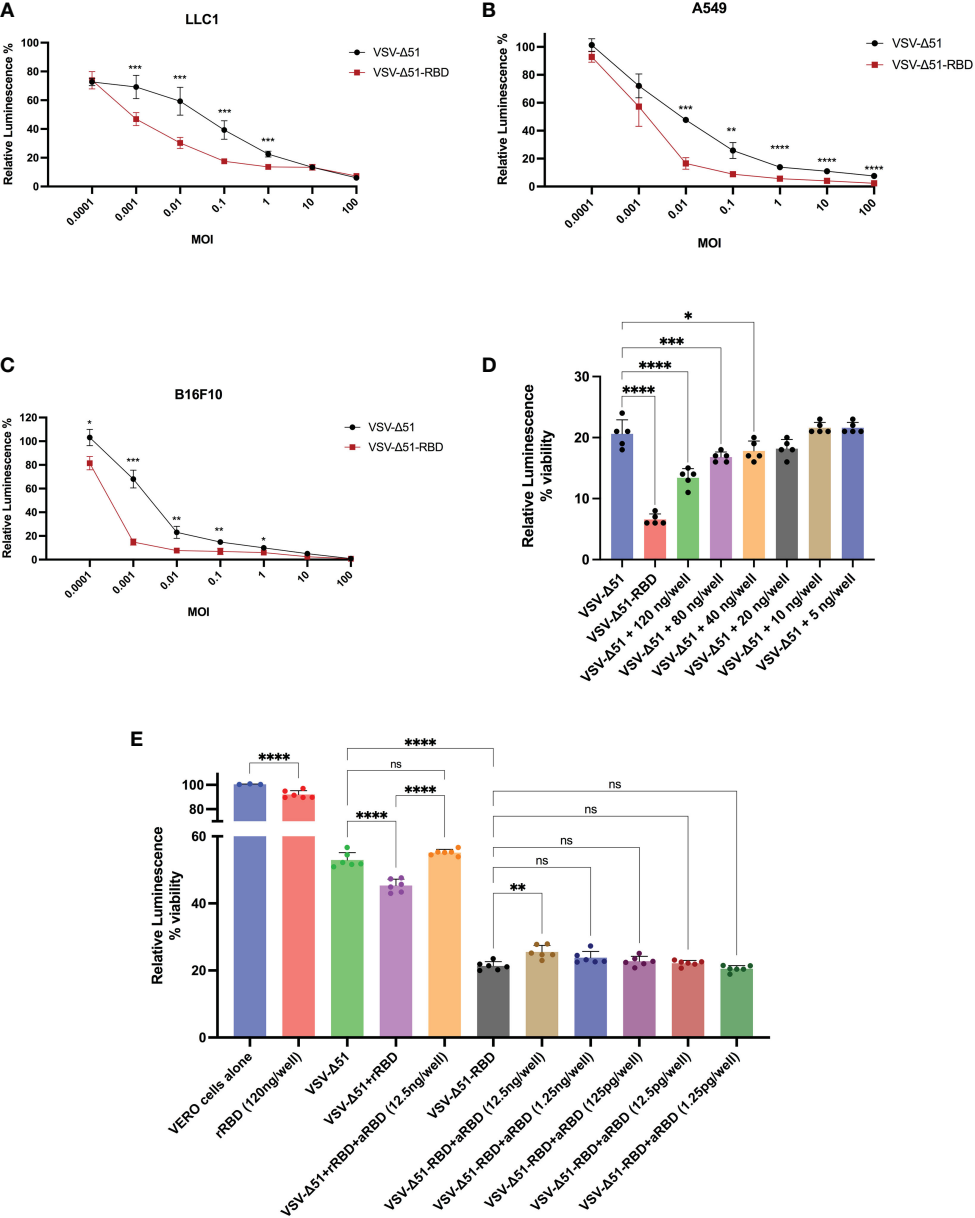
The RBD enhances the oncolytic activity of the VSV-Δ51 virus in different cancer cell lines *in vitro*: The cytotoxicity of VSV-Δ51 and VSV-Δ51-RBD was assessed in **(A)** LLC1, **(B)** A549 and **(C)** B16F10 cells at the indicated MOIs 48 hours after infection using the CellTiter-Glo^®^ viability assay. **(D)** The cytotoxicity of VSV-Δ51 was assessed in VERO cells pretreated with ascending concentrations of the recombinant RBD protein and infected with VSV-Δ51 at an MOI of 0.01. **(E)** The cytotoxicity of VSV-Δ51-RBD preincubated with different dilutions of anti-RBD neutralizing monoclonal antibodies was assessed at an MOI of 0.01 in VERO cells. “ns”, not significant, P < 0.05, *P < 0.05, ***P < 0.0005, ****P < 0.00005 by t test. ** P < 0.005.

To determine the therapeutic potential of the SARS-CoV-2 RBD, we evaluated the cytotoxicity of the VSV-Δ51-RBD and VSV-Δ51 viruses using three different cancer cell lines (LLC1 – a murine lung cancer cell line, A549 – a human lung cancer cell line and B16F10 – a murine melanoma cell line). Based on data from the CellTiter-Glo^®^ Luminescent cell viability assay, we demonstrated that the *in vitro* cytotoxicity of the VSV-Δ51-RBD virus was significantly higher (*P < 0.05) than that of the VSV-Δ51 virus at low MOIs (0.001, 0.01 and 0.1) (**Figure 3**). To further validate the contribution of the SARS-CoV-2 RBD to this phenomenon, we examined the cytotoxicity of the VSV-Δ51 virus in the VERO cell line in the presence of exogenous recombinant RBD protein. In alignment with the cytotoxicity results for the VSV-Δ51-RBD virus shown in (**Figure 3**), the addition of a recombinant RBD protein at doses of 120 ng/well, 80 ng/well and 40 ng/well significantly enhanced the cytotoxicity of VSV-Δ51 virus in a dose-dependent manner compared to infection with VSV-Δ51 alone but to a limited level compared to VSV-Δ51-RBD (*P < 0.05, ***P < 0.0005, ****P < 0.00005, respectively) (**Figure 3**). This finding is supported by the observation that the addition of anti-RBD neutralizing antibody to the culture at different dilutions abrogated the effect of the recombinant RBD protein only at a high concentration; 12.5ng/well (*P < 0.005). Interestingly, the addition of anti-RBD neutralizing antibody to the VSV-Δ51-RBD, even at a high concentration, did not completely eliminate the cytotoxicity of the VSV-Δ51-RBD to the same level as the VSV-Δ51 virus, suggesting that the RBD-enhanced toxicity is more pronounced when the RBD protein is expressed within the cytoplasm rather than when supplemented extracellularly. We also found that the recombinant RBD alone has limited cytotoxicity compared to uninfected cells (~ 93%). This suggests that RBD alone does not have strong cytotoxic activity, and its enhancement when expressed within the VSV genome is likely due to its ability to enhance viral replication and spread rather than direct cytotoxicity. These findings collectively support the potential utility of SARS-CoV-2 RBD, incorporated into the viral genome, for enhancing viral cytotoxicity.

### The SARS-CoV-2 RBD-mediated improvement of VSV-Δ51 activity could lead to better treatment outcomes

**Figure 4:**
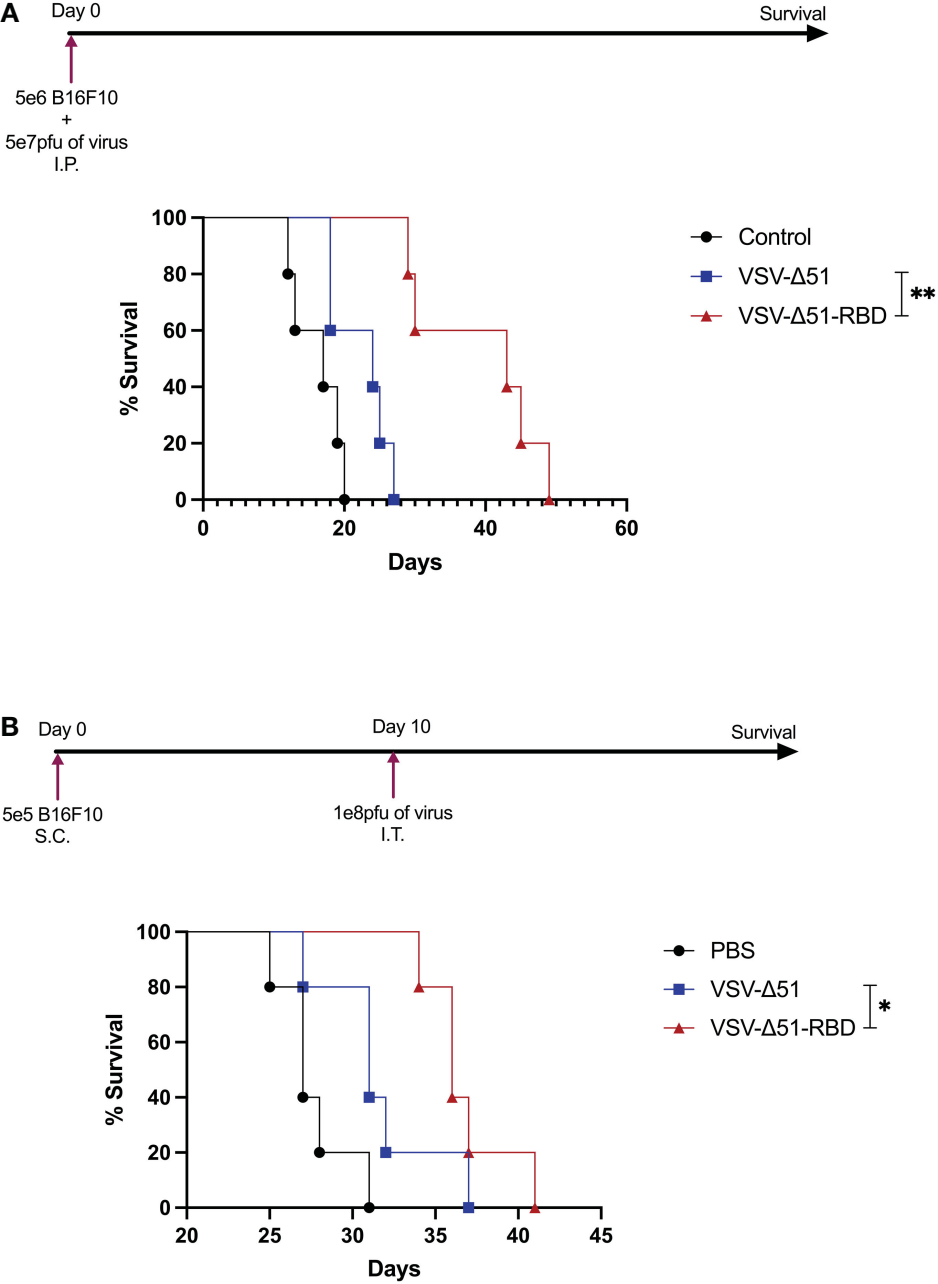
The VSV-Δ51-RBD improved treatment outcomes in B16F10 tumor models: **(A)** Kaplan–Meier survival analysis with statistics examined by a log-rank test where P < 0.05. N = 5 per group for C57BL/6 mice injected with VSV-Δ51 or VSV-Δ51-RBD-infected cells intraperitoneally. **(B)** Kaplan–Meier survival analysis of C57BL/6 mice bearing B16F10 subcutaneous tumors treated once with either VSV-Δ51 or VSV-Δ51-RBD on day 10. P < 0.05, *P < 0.05, **P < 0.005.

To evaluate the oncolytic activity of VSV-Δ51-RBD *in vivo*, B16F10 cells infected with either VSV-Δ51 or VSV-Δ51-RBD. These virus-infected cells were injected into the peritoneal cavity of C57BL/6 mice. Mice injected with VSV-Δ51-RBD-infected B16F10 cells had a significantly improved survival benefit compared to mice injected with VSV-Δ51-infected B16F10 cells (**P < 0.01) (**Figure 4A**). To further test the efficacy of VSV-Δ51-RBD in a clinically relevant tumor model, we established subcutaneous B16F10 tumors and mice were then subsequently treated with a single dose of either VSV-Δ51 or VSV-Δ51-RBD intratumorally 10 days after tumor implantation. Overall, mice treated with either VSV-Δ51 or VSV-Δ51-RBD had a significantly improved survival outcome compared to those receiving no treatment, with a median survival of 31 days and 38 days post-tumor seeding, respectively (**Figure 4B**). Even though there was a statistically significant difference between the VSV-Δ51 and VSV-Δ51-RBD treatments (*P < 0.05), there was no complete tumor remission in the VSV-Δ51-RBD-treated group. Altogether, these observations further support the therapeutic potential of the SARS-CoV-2 RBD.

## Material and methods

### Cell lines

A549 human lung carcinoma and VERO cell lines were generous gifts from Mr. Suhail Melibary (King Abdulaziz University Hospital, Jeddah, Saudi Arabia). The LLC1 and B16F10 murine cell lines were generous gifts from Dr. Nada Zaidan and Dr. Khalid Shah (KACST-BWH Centre of Excellence for Biomedicine, Riyadh, Saudi Arabia). All cell lines were cultured in Dulbecco’s modified Eagle’s medium containing 10% fetal bovine serum (Sigma Aldrich, Saudi Arabia).

### Viral titration and viral plaque assay

VERO cells were seeded into 6-well plates at a density of 5 × 10^5^ cells per well. The next day, cell monolayers were infected with 500 μl of serially diluted viruses in serum-free DMEM for 1 hour. After viral adsorption by the cells, the infectants were removed, and 2 ml of 1% agarose overlay was added (2X Dulbecco’s modified Eagle’s medium and 20% FBS: 2% agarose at a 1:1 ratio). At 48 hours post-infection (hpi), the cells were fixed with 4% formaldehyde and stained with crystal violet. Plates were washed with tap water, dried and scanned using a flatbed office scanner at a resolution of 300 dots per inch (dpi). Plaques were counted, and the surface area was measured using ViralPlaque (LowRes method, single-well mode and manual control option on). All false positive plaques were eliminated manually. Finally, the area (in pixels squared) was transformed into diameter units (mm) assuming circular plaques.

### Viruses

The VSV-Δ51-RBD virus was created in Alkayyal’s and Mahmoud’s labs by inserting a codon-optimized RBD gene, containing the amino acid region (319-541aa) of the full Spike gene of the SARS-CoV-2 (2019-nCoV), from the RBD expression plasmid (Sino Biological Inc., Beijing, China, cat# VG40592-UT) into a plasmid encoding the VSV-Δ51 antigenome plasmid, which was a gift from Dr. Anwar Hashim (King Abdulaziz University, Jeddah, Saudi Arabia). The insertion was between the G and L genes, and primers used for this insertion as follows: Forward 5’-TGGAAAGTAAGCTAGCTGTATGAAAAAAACTCATCAACAGCCATCATGAGGGTCCAACCA-3’ and Reverse: 5’-GAAGAATCTGGCTAGCTCAGAAGTTCACACACTTGTTC-3’. These primers were designed to be compatible with the In-Fusion^®^ HD Cloning Kit (Takara Bio Inc, USA, cat# 638910). Both viruses were rescued in Alkayyal’s and Mahmoud’s labs as described elsewhere (16), and were grown in VERO cells by infecting cells at 0.1 MOI and subsequently purified from the supernatant by a sucrose gradient. Virus pellets were resuspended in PBS, aliquoted and kept at –80°C until used.

### Viral cytotoxicity/cell viability

The A549, LLC1, B16F10 or VERO cell lines were seeded into 96-well plates at a density of 2 × 10^4^ cells/well. The next day, cells were infected with either VSV-Δ51 or VSV-Δ51-RBD at various MOIs ranging from 0.0001–100 pfu/cell. For the assessment of the recombinant RBD effects on the VSV-Δ51 cytotoxicity, VERO cells pretreated with ascending concentrations of the recombinant RBD protein (Sino Biological Inc., Beijing, China, cat# 40592-VNAH) as indicated in (**Figure 3D**) and infected with VSV-Δ51 at 0.01 MOI. When needed, the neutralizing anti-RBD antibodies (Acrobiosystems Inc, Newark, USA, cat# SPD-M128) were preincubated with VSV-Δ51-RBD or the recombinant RBD protein for one hour at 37°C, then added to VERO cells. Following a 48-hour incubation, CellTiter-Glo^®^ Luminescent Cell Viability Assay reagent (Promega, cat# G7571) was used to quantify the amount of ATP present in the cultures, which is reflective of the number of metabolically active cells. Briefly, 100 µl of CellTiter-Glo^®^ reagent was added to each well and incubated at room temperature for 12 minutes, two minutes of which were on an orbital shaker to induce cell lysis.

### *In vivo* cytotoxicity and therapeutic model in B16F10 cells

B16F10 tumor cells were harvested from tissue culture plates, resuspended in PBS at a concentration of 1 × 10^8^ cells/ml, and 200 μl was aliquoted into Eppendorf tubes. Cells were then infected with VSV-Δ51 or VSV-Δ51-RBD at an MOI of 10 followed by incubation for 2 hours at 37°C. Next, 100 μl of the infected cells was injected into mice intraperitoneally. Therefore, each dose contained 5 × 10^6^ B16F10 cells infected with 5 × 10^7^ pfu of the virus. The survival of the mice was monitored, and the endpoint was defined as when peritoneal distension developed. For the subcutaneous tumor model, tumors were established by injecting 5 × 10^5^ B16F10 cells in 100 µl PBS subcutaneously. Treatment began on day 10 after tumor implantation, with a single injection of VSV-Δ51 or VSV-Δ51-RBD viruses at a concentration of 1 × 10^8^ pfu.

### Statistical analysis

All statistical analyses were performed using GraphPad Prism 9.0 software. The Student’s t-test was used to determine statistical significance, with a cutoff of P = 0.05. Data are presented as ± SD. For the survival analysis, Kaplan-Meier survival curves were used and statistical analysis was performed using the Log-rank (Mantel-Cox) test, with a cutoff of P = 0.05.

## Discussion

The oncolytic activity of naturally occurring and genetically engineered viruses has been widely reported based on the replication capacity and immunogenicity of the viruses (18–20). In fact, since the mid-1800s, many case reports have documented tumor regression in patients who coincidently acquired viral infections (21–24). One of the first cited case reports was based on a 42-year-old patient who suffered from myelogenous leukemia and went into remission after what was later thought to be an influenza virus infection (25). Additionally, a recent case report based on a 20-year-old patient who suffered from a relapsed/refractory NK/T-cell lymphoma showed that the patient had a transient regression concomitant with COVID-19 onset, suggesting the oncolytic activity of the SARS-CoV-2 virus (26). Furthermore, *Oi Kuan Choong et al.* demonstrated in a preliminary study that SARS-CoV-2 displayed an oncolytic activity in papillary renal cell carcinoma (27).

Given the safety and efficacy profiles of the oncolytic virus VSV-Δ51, our group repurposed the VSV-Δ51 platform to develop a COVID-19 vaccine (our unpublished data). Surprisingly, we noticed improved viral kinetics in our COVID-19 vaccine candidate (VSV-Δ51-RBD) when compared to the parental virus, suggesting the potential of VSV-Δ51-RBD as an oncolytic virotherapy agent. In this study, we demonstrated a novel way to use the SARS-CoV-2 RBD protein to enhance VSV-Δ51 viral replication and cytotoxicity in several cancer cell lines. To further validate our findings, we used the B16F10 tumor model, which has been reported to resist viral infection (28). Interestingly, we noticed a significant improvement in the overall tumor-free survival benefit in the VSV-Δ51-RBD group compared to the VSV-Δ51 group (**Figure 4A**). This finding was also validated in the B16F10 subcutaneous model (**Figure 4B**), suggesting that the observed SARS-CoV-2 RBD efficacy is in part due to the improved viral toxicity. However, the immunogenicity of the SARS-CoV-2 RBD protein has not yet been elucidated in the context of antitumor immune responses, although this might play a role in its reported efficacy. Further investigation is needed to shed light on the lymphocyte recruitment to the site of infection following VSV-Δ51-RBD treatment.

Qi Zhang et al. demonstrated that the SARS-CoV-2 S protein, specifically the S1 region containing the RBD protein, can interfere with and downregulate the type I interferon (IFN-I) production and signaling pathway by suppressing the phosphorylation and nuclear translocation of STAT1 and disrupt its interaction with JAK1 (29). Despite this pathogenic characteristic, the proposed therapy utilizing the intrinsic type I interferon sensitivity of the VSV-Δ51 virus was found to be safe. Our observation that the overexpression of SARS-CoV-2 RBD within tumor cells of various origins may increase their sensitivity to OV infection highlights the utility of the VSV-Δ51 virus in the treatment of a broad range of resistant cancers. In addition, our demonstration that the SARS-CoV-2 RBD enhances the growth of VSV-Δ51 suggests that the employment of SARS-CoV-2 RBD will improve viral production at a large scale, which has both economic and industrial importance. However, further studies are still needed to evaluate the safety of this strategy and to illustrate the specific molecular and immune mechanisms by which the SARS-CoV-2 RBD augments the cellular machinery to produce more viral progeny. These findings could be particularly relevant for VSV-Δ51 OV.

## Data availability statement

The raw data supporting the conclusions of this article will be made available by the authors, without undue reservation.

## Ethics statement

The animal study was reviewed and approved by The Institutional Animal Care and Use Committee under project number EXO-RYD-22-419837-1645, King Abdullah International Medical Research Center, Riyadh, Saudi Arabia.

## Author contributions

AAA and AM contributed to the conception and design of the study. TA, MAA, and FK performed the *in vivo* experiments. MC, AeAA, and NS performed experiments and statistical analysis. AAA, AM, and RA wrote the first draft of the manuscript. MC, NZ, FA and KS wrote sections of the manuscript. All authors contributed to the article and approved the submitted version.
